# Empowering Extracellular Vesicle Wound Therapy via Local Drug Delivery Systems: Mechanistic Insights and Advanced Stimuli-Responsive Strategies

**DOI:** 10.3390/gels12070642

**Published:** 2026-07-18

**Authors:** Ziqiao Zhong, Ziyi Feng, Yawen Huang, Zhenhao Li, Libing Lu, Lu Gan, Xincheng Lin, Xiaolu Xiao, Yichun Zheng, Xin Pan, Chuanbin Wu, Ying Huang, Wenhao Wang

**Affiliations:** 1 State Key Laboratory of Bioactive Molecules and Druggability Assessment, Guangdong Basic Research Center of Excellence for Natural Bioactive Molecules and Discovery of Innovative Drugs, Jinan University, Guangzhou 511443, China; zhongzq@stu2022.jnu.edu.cn (Z.Z.); huangyw34@163.com (Y.H.); licho@stu2024.jnu.edc.cn (Z.L.); llb2024@stu2024.jnu.edu.cn (L.L.); 17889945251@163.com (L.G.); lxc18307544739@stu2024.jnu.edu.cn (X.L.); lucylu62025@163.com (X.X.); zyc5210140512@163.com (Y.Z.); chuanbinwu@jnu.edu.cn (C.W.); 2 College of Pharmacy, Jinan University, Guangzhou 511443, China; 3 School of Stomatology, Jinan University, Guangzhou 510632, China; fengziyi@stu2022.jnu.edu.cn; 4 School of Pharmaceutical Sciences, Sun Yat-Sen University, Guangzhou 510006, China; panxin2@mail.sysu.edu.cn

**Keywords:** extracellular vesicles, wound healing, hydrogel, drug delivery system, regenerative medicine

## Abstract

Extracellular vesicles have emerged as promising cell-free therapeutic agents for wound healing due to their remarkable ability to modulate inflammatory responses, promote angiogenesis, and enhance tissue regeneration. These biological nanocarriers deliver bioactive cargo, including regulatory miRNAs, proteins, and lipids, to recipient cells, thereby modulating key signaling pathways governing tissue repair. However, the clinical translation of extracellular vesicle (EV)-based therapies is substantially limited by challenges in delivery efficiency. Local drug delivery systems (LDDSs) offer several key advantages, including reduced clearance by the reticuloendothelial system, enhanced biodistribution to wound sites, prolonged local residence time, and precise spatial targeting of therapeutic effects. This review systematically summarizes recent advances in EV-based therapies for wound repair, with a particular focus on in situ forming and implantable LDDSs, such as stimuli-responsive hydrogels. We comprehensively discuss the molecular and cellular mechanisms through which EVs facilitate healing across all phases of wound repair. Furthermore, we critically evaluate the evolution of these delivery platforms, transitioning from conventional passive-release systems to advanced stimuli-responsive hydrogels and microneedle systems, assessing their design rationale and integration with EV biology. We also address key translational challenges and opportunities: scalable manufacturing, standardized quality control, and regulatory pathways, offering a forward-looking view on clinical implementation of EV-LDDS hybrids in precision regenerative therapy.

## 1. Introduction

The human skin functions as a critical protective barrier, dynamically maintaining homeostasis by separating the internal milieu from the external environment. While endowed with robust intrinsic repair capacity, cutaneous wound healing becomes markedly impaired upon injury, particularly in cases of compromised physiology, rendering the process inherently complex and often protracted [1].

Conventional wound management strategies, including debridement, primary closure, and antimicrobial dressings, are primarily aimed at infection control and provisional tissue support. Yet these approaches demonstrate limited efficacy for chronic or complex wounds, such as diabetic foot ulcers and full-thickness burns. Although surgical interventions like autologous skin grafting remain standard for extensive tissue loss, they are constrained by donor-site morbidity, delayed re-epithelialization, and substantial rates of graft failure and secondary complications. Stem cells have occupied a central position in regenerative medicine owing to their capacity for tissue repair and functional regeneration. Nevertheless, their clinical translation remains substantially constrained by intrinsic biological limitations, most notably, allogeneic immune rejection, unpredictable differentiation trajectories leading to teratoma formation or ectopic tissue growth, and practical hurdles related to cryopreservation stability and batch-to-batch variability. In response, extracellular vesicles (EVs), nanoscale (30–150 nm) structures actually secreted by diverse cell types, especially mesenchymal stem cells (MSCs), have emerged as highly promising acellular therapeutics. As endogenous nanocarriers, EVs inherit the regenerative signaling repertoire of their parent cells while eliminating critical safety liabilities associated with whole-cell administration. Their phospholipid bilayer membrane confers exceptional biostability and protects cargo integrity during systemic circulation or local delivery. Crucially, EVs deliver functionally coordinated bioactive molecules, including regulatory microRNAs, immunomodulatory proteins, and lipid mediators, into recipient cells primarily through receptor-mediated endocytosis and membrane fusion [2,3]. This targeted cargo transfer orchestrates a cascade of downstream effects: suppression of excessive inflammation, spatiotemporally controlled angiogenesis, balanced fibroblast activation and migration, regulated collagen synthesis and crosslinking, and dynamic remodeling of the extracellular matrix [4,5,6]. Importantly, EV-based interventions recapitulate the therapeutic benefits of stem cells, such as paracrine stimulation of endogenous repair mechanisms, without introducing replicating cells or genomic instability risks [7,8]. Accumulating preclinical and early clinical evidence demonstrates that EVs actively modulate every canonical phase of wound healing: from initiation and resolution of inflammation, through granulation tissue formation and re-epithelialization, to final maturation and scar modulation. These attributes collectively position EV therapy as a rigorously translatable, next-generation regenerative strategy with strong mechanistic grounding and escalating clinical validation.

Despite these advantages, systemic delivery, especially intravenous administration, remains suboptimal due to rapid clearance by the reticuloendothelial system, poor biodistribution to wound sites, low EV retention, and off-target effects. Consequently, localized, sustained delivery platforms are essential to harness the full therapeutic potential of EVs. Local drug delivery systems (LDDSs) represent a paradigm shift in EV therapeutics: they enable spatiotemporally controlled release directly at the wound bed, thereby maximizing local bioavailability while minimizing systemic exposure and toxicity. Beyond controlled release, LDDSs serve dual functional roles, acting both as protective carriers that preserve EV integrity and bioactivity and as biomimetic 3D scaffolds that provide mechanical cues and structural guidance for infiltrating cells, synergistically amplifying regenerative outcomes. Recent years have witnessed a paradigm shift in EV-mediated wound therapy, marked by the emergence of multi-stimuli-responsive delivery systems, biomimetic nanocomplexes, and scalable manufacturing approaches. Recent advances include the development of sprayable EV-loaded hydrogels with controlled release for diabetic wound healing [9], core–shell microneedle patches integrating reactive oxygen species (ROS)-responsive and EV-laden layers for spatiotemporal therapy [10], and oxygen-generating hydrogels that simultaneously alleviate hypoxia and deliver EVs [11]. These innovations underscore the field’s trajectory towards more sophisticated, personalized, and clinically translatable solutions.

**Figure 1:**
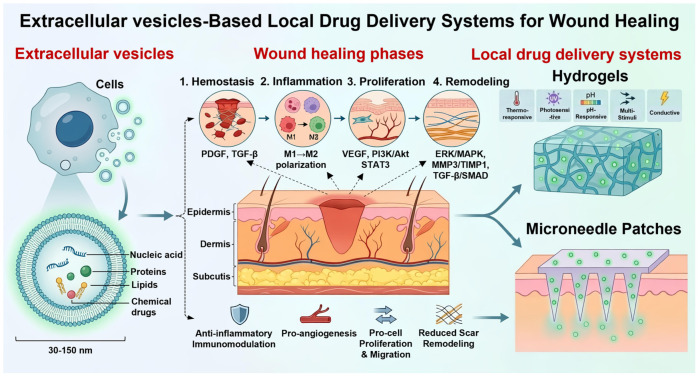
An EV-based local drug delivery system for wound healing, along with the associated therapeutic processes and underlying molecular mechanisms.

In this review, we critically evaluate recent advances in EV-integrated wound healing platforms. First, we delineate the molecular and cellular mechanisms underpinning EV-mediated tissue repair. Second, we comprehensively assess kinds of LDDSs, including conventional hydrogels, stimuli-responsive hydrogels and microneedles, with emphasis on their design rationale, release kinetics, and functional integration with EV biology (Figure 1). Finally, we address translational challenges and opportunities in scalable EV manufacturing, quality control, and regulatory standardization, providing a forward-looking perspective on the clinical implementation of EV-LDDS hybrids in precision regenerative therapy. In contrast to existing reviews on EVs, this review places particular emphasis on the rational design and development of advanced LDDSs informed by mechanistic insights into wound healing and tissue repair. Furthermore, it critically examines key challenges impeding the clinical translation and scalable manufacturing of EV-based therapeutics. Although “exosomes” is widely used in the literature, the biogenesis of vesicles isolated from conditioned media is often unconfirmed. Following the International Society for Extracellular Vesicles guidelines (MISEV), we use the term “EVs” throughout this review, reserving “exosomes” only for studies that have definitively demonstrated endosomal origin.

## 2. Mechanisms of Wound Healing

Any disruption to the integrity of living tissue can be deemed a wound. In 2014, Medicare expenditures for acute and chronic wound care were estimated to range from $28.1 billion to $96.8 billion. Surgical wounds represented the largest portion of these costs, followed closely by diabetic foot ulcers. Acute wounds, usually caused by trauma, cuts, or burns, possess a clear healing phase and are typically repaired within a few weeks. Surgical wounds are also acute wounds and generally heal within the expected time frame unless complications such as infection occur. However, certain conditions, such as diabetes, can transform acute wounds into chronic wounds due to insufficient local blood supply and low immune response. For example, diabetic foot ulcers usually present as persistent or recurrent injuries that are difficult to heal and may last for months or even longer.

**Figure 2:**
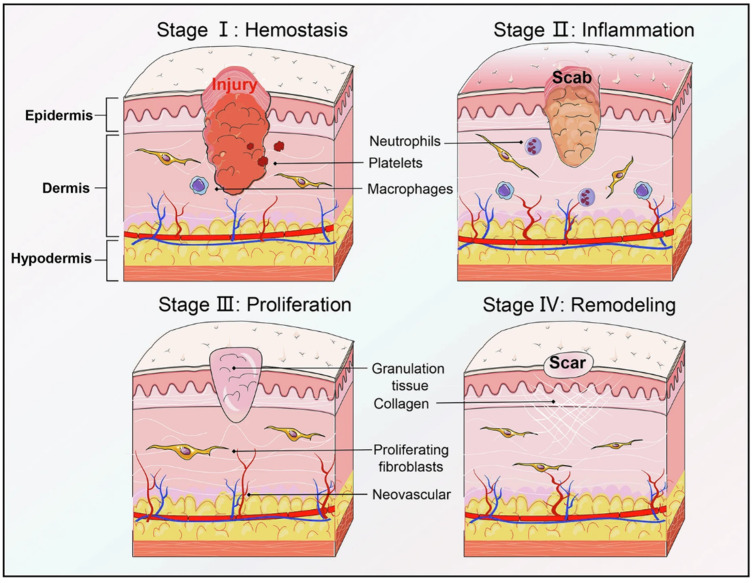
The process of wound healing and scarring. Wound healing is a highly coordinated physiological process aimed at restoring tissue integrity and functional capacity following injury. It proceeds through four temporally overlapping yet functionally distinct phases: hemostasis, inflammation, proliferation, and remodeling. Each phase is tightly regulated and involves the dynamic interaction of multiple cell types, including fibroblasts, keratinocytes, endothelial cells, and immune cells, as well as extracellular matrix components and signaling molecules. Disruptions to this process, such as those caused by infection, chronic hyperglycemia, or autoimmune disorders, can impair progression through one or more phases, thereby delaying wound closure and increasing the risk of pathological scar formation. Adapted from Qi Zhang et al. Reference [16] under the terms of the Creative Commons CC BY license.

Cutaneous wound healing proceeds through four overlapping, tightly coordinated phases: hemostasis, inflammation, proliferation, and remodeling (Figure 2). Disruption of spatiotemporal regulation across any phase, particularly prolonged inflammation or failed transition to proliferation, predisposes to chronicity. Hemostasis is the earliest and most transient phase, initiated immediately upon injury: platelet adhesion and aggregation at the wound site trigger thrombin generation and release of bioactive mediators, including PDGF, TGF-β, and serotonin, that recruit fibroblasts and stimulate early collagen synthesis [12]. The subsequent inflammatory phase, marked by vasodilation, increased vascular permeability, neutrophil infiltration, and macrophage polarization, serves not only to clear debris and pathogens but also to orchestrate the transition to proliferation via cytokine-mediated crosstalk [13]. Beginning approximately 72–96 h post-injury and peaking between days 4–7, the proliferative phase is characterized by robust keratinocyte migration (re-epithelialization), fibroblast proliferation, angiogenesis, and granulation tissue formation, which is the provisional matrix that restores barrier function and provides structural support [14]. Finally, the remodeling phase extends over months to years: during this period, type III collagen is progressively replaced by mechanically superior type I collagen; myofibroblasts mediate wound contraction through actin–myosin-driven tension and regulate extracellular matrix (ECM) homeostasis by balancing secretion and degradation of ECM components, including controlled activation of matrix metalloproteinases and their endogenous inhibitors [15]. Thus, wound healing is not a linear sequence but a dynamic, feedback-regulated cascade wherein precise temporal coordination among cellular effectors, soluble signals, and matrix cues is essential for timely resolution, and its dysregulation underpins all forms of impaired healing.

## 3. EVs in Wound Healing

The concept of extracellular vesicles dates back to the 1980s when they were first observed during the maturation of sheep reticulocytes [17]. Initially considered as “dust” or cellular debris responsible for discarding transferrin receptors, these vesicles were later termed “exosomes,” and their biological significance has since been extensively redefined [18]. Over time, the field of EV research has rapidly evolved, and EVs have been found to have important functions in a variety of biological regenerative and repair processes, including intercellular signaling and immunomodulation. Several studies have confirmed that EVs play an important role in the treatment of various skin injuries. In this article, we discuss the use of EVs in several deficiencies within the phases of wound healing. One of the key benefits of EV therapy lies in its ability to circumvent potential risks in comparison to stem cell therapy [4], including the reduction in immune rejection and potential heterogeneous reactions. Additionally, EVs have the advantage of easier storage and application [5]. In addition, their unique lipid bilayer membrane structure can protect their contents and resist environmental damage [6]. EVs have been considered an advanced strategy by providing regeneration-promoting therapeutic effects similar to those of the producing cells and have shown great promise for clinical applications in regenerative medicine [7,8].

### 3.1. The Molecular Mechanism of EVs in Wound Healing

Tissue repair and regeneration are orchestrated by the binding of growth factors to specific cell-surface receptors, thereby triggering key cellular responses, including cell migration, angiogenesis, re-epithelialization, extracellular matrix deposition, and tissue remodeling. Emerging evidence indicates that EVs actively modulate all stages of wound healing. EVs exert multifaceted therapeutic effects. For example, they attenuate excessive inflammation, stimulate angiogenesis, enhance cell migration and proliferation, promote collagen synthesis, and guide extracellular matrix remodeling to reduce pathological scarring. A growing body of research has elucidated the molecular mechanisms underlying the anti-inflammatory and immunomodulatory functions of EVs in wound repair. For instance, mesenchymal stem cell-derived exosomes have been shown to suppress B- and T-lymphocyte proliferation and downregulate pro-inflammatory cytokines such as TNF-α and IL-6. Furthermore, these exosomes promote macrophage polarization toward the anti-inflammatory M2 phenotype by reducing PKNOX1 expression in macrophages, thereby dampening the local inflammatory response [19,20].

Importantly, EVs stimulate the proliferation and migration of various cell types essential for wound healing, particularly fibroblasts. As the predominant cells in dermal tissue, fibroblasts are activated by injury and subsequently undergo proliferation, migration, and differentiation to synthesize extracellular matrix components, such as collagen and elastic fibers, thereby promoting tissue repair. EVs upregulate the expression of collagen type I and collagen type III genes and promote fibroblast proliferation and collagen synthesis, facilitating skin wound healing. EVs from adipose-derived mesenchymal stem cells, through the PI3K/Akt signaling pathway, can promote fibroblast proliferation, migration, collagen synthesis, and secretion of other growth factors, thereby promoting skin wound healing [21].

Wound healing is a metabolically demanding process that necessitates increased delivery of oxygen and essential nutrients. Consequently, robust neovascularization, and particularly the stimulation of angiogenesis, is critical to support tissue regeneration, as newly formed blood vessels serve as conduits for oxygen, nutrients, and immune cells to the wound site. Substantial evidence indicates that EVs carry a rich cargo of pro-angiogenic proteins and regulatory RNAs. Upon uptake by endothelial cells, these EV-associated molecules activate key signaling pathways, including PI3K/AKT, MAPK/ERK, and Notch, thereby enhancing endothelial proliferation, migration, and tube formation, and ultimately promoting functional neovascularization within the wound bed [22,23]. Moreover, mesenchymal stem cell-derived exosomes have been shown to activate the STAT3 signaling pathway, upregulate expression of cell cycle-associated genes, including c-MYC and cyclins, and encode multiple paracrine mediators such as interleukin-6, hepatocyte growth factor, stromal cell-derived factor-1, and vascular endothelial growth factor. Collectively, these molecular actions coordinate distinct phases of angiogenesis and extracellular matrix remodeling [24]. Recent studies have uncovered additional mechanistic layers. For instance, exosomes derived from 3D-cultured mesenchymal stem cells were shown to be enriched in miR-150-5p, which promotes corneal epithelial and stromal cell proliferation while suppressing inflammatory cytokine release by targeting PDCD4 [25]. In diabetic wound models, EVs have been demonstrated to regulate glucose metabolism within the wound microenvironment, representing a dual-action strategy that addresses both hyperglycemia and impaired healing [26]. Furthermore, EV-coated oxygen nanobubbles have been engineered to enhance intracellular cargo delivery under hypoxic conditions, overcoming a major barrier in chronic wound therapy [11].

EVs inhibit myofibroblast aggregation, reducing scar formation, modulating signaling pathways and limiting excessive skin fibroblast expansion and collagen deposition. EVs regulate ECM remodeling for scarless skin healing by stimulating the ERK/MAPK pathway in dermal skin fibroblasts and upregulating the ratio of MMP3 to TIMP1 [27]. Jiang et al. found that hBM-MSC-derived exosomes could inhibit the TGF-β1/SMAD signaling pathway, thus inhibiting myofibroblast differentiation and scar formation [28]. Various types of cell-derived EVs, including mesenchymal stem cells, platelets, and endothelial cells, have been reported to promote wound healing. Compared with cell therapy, extracellular vesicle therapy carries a lower risk of tumorigenicity, induces less immune response, and more effectively stimulates fibroblast expansion and migration. The key challenge lies in how to maximize the efficacy of EVs, and optimizing the biomaterial composition forms the crux. Furthermore, exploring methods for enhancing the targeting and retention of these vesicles at wound sites will greatly impact their therapeutic potential in clinical applications.

### 3.2. Standardization and Quality Control of Therapeutic EVs

The clinical translation of EV-based wound therapies is critically dependent on the reproducibility of EV production and the rigorous characterization of their physicochemical and biological properties. In accordance with the MISEV guidelines, a comprehensive evaluation encompassing source cells, isolation methods, and multiparametric characterization is essential [29].

#### 3.2.1. Source Cells and Culture Conditions

The biological cargo and therapeutic efficacy of EVs are inherently dictated by their parental cells. For wound healing applications, MSCs, particularly those derived from adipose tissue (ADSCs), bone marrow (BMSCs), and umbilical cord (hUCMSCs), are the most widely utilized source cells due to their robust paracrine signaling, immunomodulatory capabilities, and angiogenic potential [30]. Additionally, EVs derived from endothelial progenitor cells, keratinocytes, and platelets are employed for phase-specific functions. It is crucial to note that the culture conditions, such as 2D/3D culture, hypoxic/normoxic preconditioning, and passage number, profoundly influence EV secretion rates and cargo loading, such as miRNA and protein profiles, necessitating stringent standardization of source cell manufacturing processes [31]. Recent advances in 3D culture systems and bioreactor technologies are enabling more scalable and reproducible production of clinical-grade MSC-EVs [32].

#### 3.2.2. Isolation Methods

The choice of isolation technique directly impacts EV yield, purity, and downstream functionality, yet no universal “gold standard” currently exists, and each method presents distinct trade-offs [33]. Ultracentrifugation, the most traditionally utilized approach, yields high quantities of EVs but subjects them to high shear forces that can compromise integrity, while the resulting pellets often co-precipitate non-EV proteins and lipoproteins, lowering purity. In contrast, density gradient centrifugation using iodixanol achieves superior purity by separating EVs based on buoyant density and effectively removing protein contaminants, albeit at the cost of being time-consuming and producing lower yields that limit scalability. Size-exclusion chromatography offers a different balance, preserving vesicle integrity and biological activity by separating based on size, and is increasingly favored for clinical applications owing to its reproducibility and ability to generate highly pure EV populations, though it requires concentration steps for dilute samples [34]. For large-scale, GMP-compliant production, tangential flow filtration stands out as highly scalable and efficient at maintaining EV integrity, yet it may struggle to separate EVs from similarly sized particles [35]. Meanwhile, emerging affinity-based methods, such as the use of meticulously engineered peptides or DNA microflowers for rapid and unbiased EV enrichment, are showing great promise for achieving high-purity isolation from complex biofluids within minutes [36,37].

#### 3.2.3. Characterization: Particle Size, Concentration, and Surface Markers

Rigorous characterization requires complementary techniques to validate EV identity. For particle size and concentration, Nanoparticle Tracking Analysis (NTA) is the standard technique for determining EV concentration (particles/mL) and size distribution (typically 30–150 nm for small EVs), while Tunable Resistive Pulse Sensing (TRPS) offers higher resolution for polydisperse samples, and Transmission Electron Microscopy (TEM) or Cryo-EM is essential for visualizing the characteristic cup-shaped morphology and confirming structural integrity [38]. Regarding surface markers, Western blotting and flow cytometry are employed to verify the presence of EV-enriched proteins; according to MISEV guidelines, characterization should include at least three positive protein markers (e.g., tetraspanins such as CD9, CD63, and CD81, and cytosolic proteins like TSG101 and Alix) alongside negative markers (e.g., endoplasmic reticulum proteins such as Calnexin) to rule out cellular contamination [39]. Moreover, advanced techniques like single-EV flow cytometry and spatial EV sequencing (e.g., Spatial-EVs-seq) are emerging as powerful tools for high-dimensional phenotyping and mapping EV heterogeneity in tissues [40].

#### 3.2.4. Purity, Protein Contamination, and Functional Potency Assays

A critical, yet often overlooked, metric in EV preparation is the assessment of purity and protein contamination, where the particle-to-protein ratio (PPR) is the recommended metric for evaluating purity, as a low PPR indicates significant co-isolated protein contaminants, such as albumin and lipoproteins, that can confound therapeutic dosing and trigger adverse immune responses [41]. Assays such as the BCA or Bradford method are used to quantify total protein, which must be correlated with particle count. Furthermore, physical characterization alone is insufficient to predict clinical efficacy, and functional potency assays tailored to the intended therapeutic mechanism are mandatory, with wound healing applications requiring that these assays quantifiably demonstrate EV bioactivity in vitro before in vivo translation [42]. Standard potency assays include proliferation and migration assays, which evaluate the EVs’ capacity to accelerate fibroblast or keratinocyte closure in scratch assays; angiogenic assays assess the ability of EVs to promote endothelial cell (e.g., HUVEC) tube formation on Matrigel [43]. Immunomodulatory assays measure the polarization of macrophages (e.g., M1 to M2 transition) via flow cytometry or evaluate the suppression of pro-inflammatory cytokines, such as TNF-α and IL-6, in LPS-stimulated cells [44]. The development of standardized, robust potency assays remains an active area of research and is critical for regulatory approval and batch-to-batch consistency.

## 4. In Situ Delivery Systems for EVs

Establishing a protective barrier around a wound plays a critical role in wound healing. A biobarrier, a physical or biological isolating structure, not only protects the wound from infection and external damage but also provides a suitable environment for maintaining proper moisture levels at the wound site to promote tissue repair and the healing process. One of the primary challenges hindering the clinical translation of EVs is the lack of suitable delivery systems. Effective EV delivery systems must meet several criteria: high targeting capability to ensure accurate and rapid delivery to the wound site; sustained release ability to maintain effective drug concentration at the wound site; and biocompatibility and biodegradability to reduce irritation and damage to surrounding tissues while gradually degrading as the wound heals. Conventional drug delivery methods such as intravenous administration fail to fully address these requirements and present significant drawbacks, including imprecise dosing regulation, reduced survival of EVs in vivo, and high risk of off-target effects. Thus, there is an urgent need for safer and more effective EV delivery systems to provide better therapeutic options for promoting wound healing. Local drug delivery systems (LDDSs) represent a more effective and safer option for wound healing, with the potential to revolutionize wound therapy. When selecting biomaterials for EV delivery, hydrogels have been most extensively investigated for tissue regeneration applications. For instance, studies have shown that MSCs and macrophages seeded into polyglycolic acid scaffolds and injected with MSC-derived exosomes can significantly improve the efficacy of myocardial regeneration [45]. Other biomaterials, such as microneedle patches, have also been employed in several studies.

**Figure 3:**
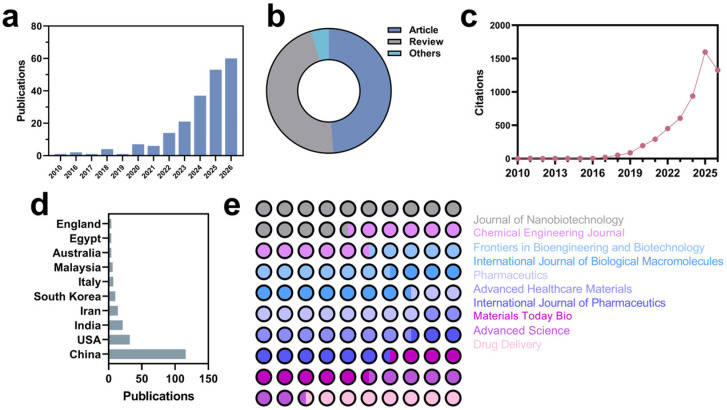
Bibliometric analysis of LDDSs used in EV delivery. (**a**) Number of publications versus year. (**b**) Types of publications. (**c**) Citations of these publications. (**d**) Numbers of publications from top 10 regions. (**e**) Number of publications in top 10 periodicals.

To investigate the development of LDDSs used in EV delivery, a literature survey was employed based on the Web of Science (Figure 3). Keywords are exosomes, wound healing and local drug delivery systems. The results demonstrated that approximately 208 publications in this field were presented in the past 5 years (Figure 3a). The year 2026 was the peak of the number of publications, and “article” was the main publication type (Figure 3b). The number of citations related to LDDSs in EV delivery has been steadily increasing in recent years, with a count of nearly 1600 citations for the year 2025 (Figure 3c). The papers published by the researchers from China accounted for 116 (Figure 3d). Additionally, the top five journals that have published papers pertaining to LDDSs in EV delivery include *Journal of Nanobiotechnology*, *Chemical Engineering Journal*, *Frontiers in Bioengineering and Biotechnology*, *International Journal of Biological Macromolecules* and *Pharmaceutics* (Figure 3e). This observation signifies a growing emphasis and interest in this field. Thus, it is necessary to summarize and analyze the current publications on LDDSs in EV delivery, enabling the scientific community to gain the latest development direction and research focus in this field more clearly.

### 4.1. Hydrogel Composites for Extracellular Vesicle Delivery

A hydrogel is a stable hydrophilic network structure that is physically, chemically and bio-enzymatically crosslinked with good degradability, water retention and biocompatibility. The swelling property of hydrogels creates a suitable moist environment for wound healing. Their porous structure facilitates absorption of wound exudates and oxygen permeation to promote wound healing [46]. Hence, it can be an appropriate candidate for Vencapsulation based on these characteristics [47].

#### 4.1.1. Conventional Hydrogels

EVs combined with hydrogels have shown promising therapeutic effects in studies related to tissue regeneration and angiogenesis. Some natural materials, such as hyaluronic acid hydrogels (HA), have been used to deliver cell-derived exosomes for tissue regeneration because of the advantage of allowing sustained and controlled release for improved bioavailability. Liu et al. designed two HA derivatives (adipic acid dihydrazide-modified HA, aldehyde-grafted HA) that could be reacted with Schiff base to form a hydrogel by in situ double syringe injection under mild neutral conditions [48]. The antibacterial effect of the complex was excellent, with a broad-spectrum antimicrobial effect better than ampicillin, even eradicating 99% of MRSA-resistant organisms. The M2 Exos release curves showed that the composite hydrogel had the sustained releasing property of M2 Exos within 21 days. In addition, a lack of oxygen supply to the wound could inhibit the healing process, resulting in vasculopathy of the wound and inhibition of angiogenesis by diabetes mellitus. Xiong et al. developed an injectable hydrogel that is both a biodegradable and biocompatible hydrogel for improving diabetic wound healing. Their addition of MnO_2_/ε-PL nanosheets in hydrogels enabled the conversion of excess H_2_O_2_ produced in wounds to O_2_ [49].

Alginate (Alg)-based hydrogels are similarly one of the representatives of natural hydrogels. They are of interest for encapsulating EVs due to their high biocompatibility, low cost and relative ease of gelling with divalent cations under mild conditions. Shafei et al. characterized the physical and biochemical properties of Alg hydrogels loaded with EXO as bioactive scaffolds wrapped around adipose-derived stem cell-derived exosomes. The data demonstrated that the prepared structures were biodegradable and biocompatible. Moreover, the cumulative release of EXO from Alg-EXO in Alg hydrogels reached more than 50% in the first 72 h, and the EXO release continued up to 172 h. Alg-EXO could shorten the healing time of rat skin wounds and reduce scar formation. Both in vitro and in vivo studies confirmed the potential of Alg-EXO for clinical application in wound healing [50].

**Figure 4:**
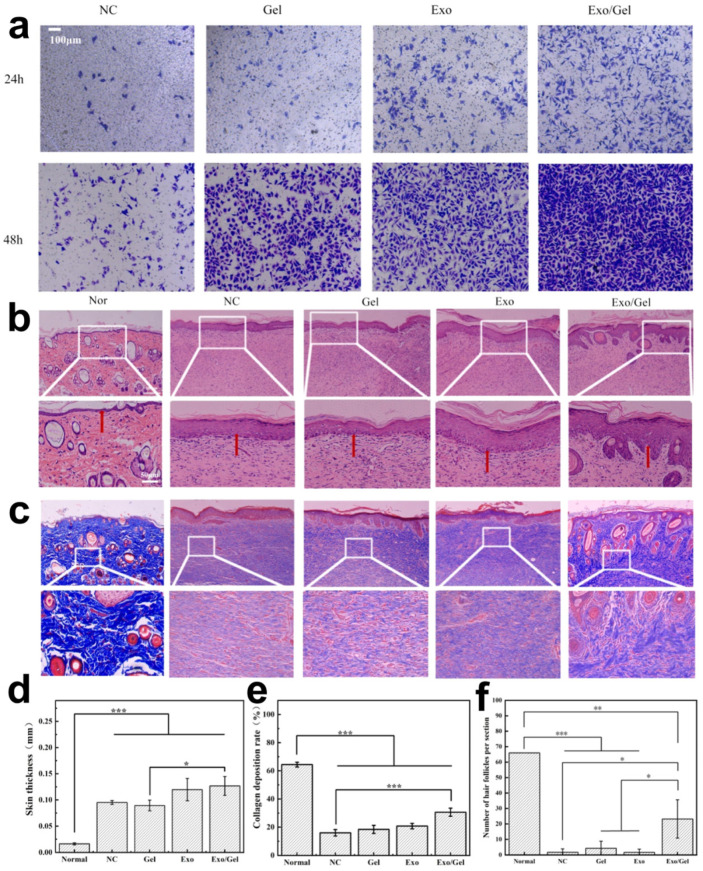
Effects of the composite hydrogel on human umbilical vein endothelial cell migration and wound tissue histomorphology. (**a**) Transwell assay images depicting HUVEC migration after 24 or 48 h under the following conditions: untreated control (NC group); treatment with 200 μL of blank hydrogel (Gel group); treatment with exosomes at a final concentration of 30 μg/mL (Exo group); and treatment with 200 μL of hydrogel loaded with 40 μg exosomes (Exo/Gel group). Scale bars: 200 μm. (**b**) Hematoxylin and eosin (H&E) staining of wound tissue sections. The red arrow indicates the thickness of the dermis. Scale bars: 200 μm. (**c**) Masson’s trichrome staining of wound tissue sections. Scale bars: 200 μm. (**d**) Quantitative analysis of dermal thickness in wound tissue sections. (**e**) Quantitative analysis of collagen deposition area fraction in wound tissue sections. (**f**) Quantitative analysis of hair follicle density (number per mm^2^) in wound tissue sections. * *p* < 0.05, ** *p* < 0.01, ***p < 0.001. Reproduced from Reference [53] with permission from American Chemical Society. Copyright © 2024, American Chemical Society.

Chronic wound infections are also considered a major reason for delayed healing, leading to recent research in the development of microneedles that can inhibit or improve wound inflammation, which is a research priority [51]. According to the literature, chitosan is the most frequently used biomaterial in this field. Chitosan is a natural cationic biopolymer with good antimicrobial properties that can be used as a wound dressing and drug delivery carrier to promote wound healing and has a broad application prospect [52]. In this study, a composite wound dressing consisting of a chitosan hydrogel and adipose-derived mesenchymal stem cell-derived exosomes (ADMSC-Exo) was fabricated via physical mixing, and its regenerative function was subsequently evaluated in a rabbit skin trauma model. Transwell experiments were performed to evaluate the ability of the exosome-loaded composite hydrogel to promote cell migration, and the data showed that Chitosan hydrogel and ADMSC-derived exosomes can synergistically promote cell migration (Figure 4a). Masson staining results showed that the Exo/Gel group had the highest density of collagen deposition and regular arrangement, which was the closest to normal skin tissues (Figure 4b,c). The Exo/Gel composite hydrogel can promote collagen deposition in skin tissues, enhance the regeneration of the dermal layer, and significantly improve wound healing (Figure 4d,e) [53]. However, the poor water solubility of chitosan is a major limiting factor for its application [54]. To address this limitation, Peng et al. designed a novel biocompatible scaffold by grafting chitosan with dihydrocaffeic acid to solve the problem of poor water solubility of chitosan and by combining the amino group of CS-DA with the aldehyde group of PF127 via Schiff base bonding to enhance the strength and self-healing ability of the hydrogel, and the anti-inflammatory ability of the hydrogel is improved by adding TA. The resulting CS-DA/PF/TA/3D-Exo hydrogel can slow-release 3D-Exo [55]. Multifunctional composite hydrogels generated based on natural polysaccharide chitosan derivatives have better rheological, self-healing, haemostatic and antimicrobial properties, and are considered to be ideal dressings for the treatment of diabetic wounds. Geng et al. reported a novel bone marrow mesenchymal stem cell-derived exosome (MSC-Exo)-loaded carboxyethyl chitosan (CEC)–dialdehyde carboxymethyl cellulose (DCMC) hydrogel (MSC-Exos@CEC-DCMC HG). As an effective therapeutic agent to synergistically modulate the inflammatory microenvironment of trauma and promote neovascularisation in type 1 diabetic rats. Intrinsic MSC-Exos not only promote angiogenesis but also the conversion of M1-type macrophages to M2-type, thereby reducing the inflammatory response and accelerating wound healing [56]. Qin et al. combined oxidized glucan with hydroxybutyl chitosan as an exosome carrier of BMSC origin using a reversible Schiff base reaction, and the composite hydrogel reduced exosome leakage by 30.4 and 42.4% at 14 and 16 h of hydrogel network disruption, respectively, compared to chitosan hydrogel alone. The hydrogel significantly enhanced the healing of stretchable wounds in a full-layer skin defect model [57].

The integration of EVs with conventional hydrogels is designed to enhance their therapeutic efficacy by prolonging release at the wound site. This prevents rapid systemic clearance and dilution, thereby maintaining an effective local drug concentration over a sustained period and reducing the frequency of administration. In addition, hydrogels form a biological barrier to protect injured skin from contamination and infection from the external environment. However, most hydrogels of natural origin do not have a stimuli-responsive function. Despite this, they still play an important role in wound management and therapy, providing patients with an effective and safe treatment option.

#### 4.1.2. Stimuli-Responsive Hydrogels

In response to the limitations of conventional hydrogels, researchers have developed various Stimuli-responsive hydrogels with enhanced capabilities for EV delivery. These advanced systems incorporate stimuli-responsive properties that enable precise control over EV release in response to specific environmental triggers.

##### Thermoresponsive Hydrogels

Thermoresponsive hydrogels hold significant promise in biomedical applications, particularly in regenerative medicine and controlled drug delivery. Pluronic F-127 (PF-127) is a clinically approved, biocompatible triblock copolymer (PEO–PPO–PEO) designated by the U.S. Food and Drug Administration to be generally recognized as safe for human use. In tissue engineering, PF-127 is widely employed as a carrier for EVs owing to its reversible thermogelling behavior: it remains a free-flowing sol at low temperatures (e.g., 4 °C) and undergoes sol–gel transition into a physically crosslinked, injectable hydrogel upon warming to physiological temperature (37 °C), thereby enabling minimally invasive, in situ delivery and sustained retention at the wound site. Recent studies have further validated the utility of PF-127 hydrogels for EV delivery. For instance, Yang et al. developed a composite hydrogel comprising PF-127 and hUCMSC-derived exosomes (hUCMSC-exos), demonstrating that the gelation temperature is tunable through modulation of PF-127 concentration [58]. Specifically, the 20% (*w*/*v*) hUCMSC-exos/PF-127 formulation exhibited gelation onset at 17.8 °C, whereas the 28% (*w*/*v*) formulation gelled at 12.4 °C; gelation time decreased with increasing temperature, consistent with the kinetics of micellar aggregation and packing. The optimized 24% (*w*/*v*) hUCMSC-exos/PF-127 formulation remained liquid at 4 °C and formed a stable, semisolid colloidal hydrogel at 37 °C. Functionally, PF-127 hydrogels provide a moist, protective wound interface that supports re-epithelialization while acting as a physical barrier against microbial invasion and environmental contaminants, contributing to significantly improved healing outcomes relative to untreated controls [58]. Critically, the synergistic combination of PF-127 and hUCMSC-exos markedly accelerated wound closure, enhanced granulation tissue formation, as evidenced by elevated expression of the endothelial marker CD31 and the proliferation marker Ki67, and upregulated key regenerative mediators, including vascular endothelial growth factor and transforming growth factor β-1, thereby coordinately promoting angiogenesis, fibroblast activation, and matrix remodeling.

In addition, the UV-shielding performance of the dressing could protect cells from damage. Polyethyleneimine (PEI) and aldolaplan (APu) were grafted onto PF-127 via a reversible Schiff base reaction to form a polysaccharide-based fluorinated ethylene propylene (FEP) scaffold dressing with significant UV-shielding properties. FEP@exos undergoes a sol–gel transition at 30 °C to continuously release EVs and maintain EV bioactivity, which significantly accelerates the proliferation and migration of endothelial cells in vitro. FEP@exos significantly accelerates endothelial cell proliferation, migration and tube formation in vitro, promoting angiogenesis and diabetic wound healing [59].

Some amphiphilic copolymers in water undergo a reversible sol–gel transition when heated. If the transition temperature is between room temperature and body temperature, the aqueous system can be readily mixed with the drug or cells at room temperature and the mixture is injectable; the injected preparation physically gels at body temperature and the gelation is free of any chemical crosslinking. Liu et al. prepared an injectable temperature-sensitive hydrogel by incorporating human umbilical cord blood-derived exosomes (UCB-Exos) into an ABA-type amphiphilic hydrogel [60]. The storage modulus (G’) of this hydrogel increases dramatically at 25 °C, indicating the formation of a stable hydrogel network. It also maintains a high concentration of exosomes at the wound site by precisely managing the multilevel hydrogen-bonding structure within the system. Data indicated that the fluorescence of exosomes increased continuously, peaked at 48 h, and then gradually decreased. The fluorescence intensity observed at 72 h corresponded to the initial measurement. This feature can be used for continuous drug delivery. Temperature-sensitive hydrogels for EV delivery, in comparison to conventional dressings, remain in a sol–gel state at low temperatures and can transform into a solid gel at appropriate temperatures. This transformation effectively retains exosomes on the wound surface, reducing their loss, and may hold significant translational value in clinical settings.

##### Photosensitive Hydrogels

Gelatin methacryloyl (GelMA) is a gelatin derivative produced by crosslinking gelatin with methacrylic anhydride in the presence of a photoinitiator using ultraviolet or visible light, possessing suitable biological characteristics and tunable physical properties akin to the extracellular matrix [61,62]. GelMA hydrogels have been widely utilized in recent studies due to their tunable mechanical properties and capacity to retain EVs for prolonged periods. Through non-covalent interaction forces and physical sequestration, Hu et al. developed anoxically pre-treated ADSC-Exo-embedded GelMA hydrogels. These materials can adapt to irregular diabetic wounds by transforming to the gel state at a rapid rate upon exposure to light. Delivery of circ-Snhg11 from GelMA-HExo enhances endothelial cell (EC) survival and increases EC migration, proliferation and vascular regeneration potential through activation of the miR-144-3p/NFE2L2/HIF1 α signaling pathway [63].

**Figure 5:**
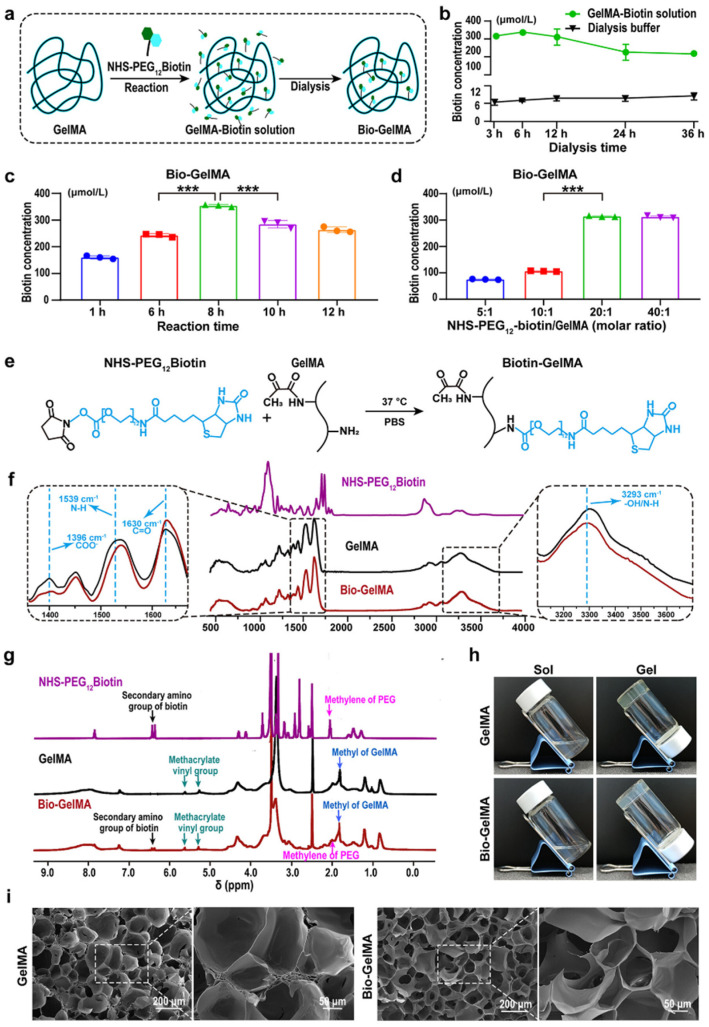
Fabrication and characterization of biotin-modified gelatin methacryloyl (Bio-GelMA) hydrogels. (**a**) Schematic representation of the covalent conjugation of NHS-PEG12–biotin to primary amine groups of GelMA to synthesize biotinylated GelMA (Bio-GelMA). (**b**) Time-dependent biotin concentration profiles in the dialysate (external solution) and retained solution (within the dialysis bag) during purification by dialysis (measured at 3, 6, 12, 24, and 36 h; *n* = 3), confirming removal of unreacted NHS-PEG12–biotin. (**c**) Bio-GelMA yield as a function of reaction time (1, 6, 8, 10, and 12 h; *n* = 3), indicating reaction progression toward completion. (**d**) Bio-GelMA yield achieved after 8 h of reaction under varying molar feed ratios of NHS-PEG12–biotin to GelMA (5:1, 10:1, 20:1, and 40:1; *n* = 3), demonstrating optimization of labeling efficiency. (**e**) Chemical reaction scheme for Bio-GelMA synthesis, illustrating nucleophilic acyl substitution between NHS–ester and primary amine functionalities. (**f**) Fourier-transform infrared (FT-IR) spectra of NHS-PEG12–biotin, GelMA, and Bio-GelMA. Left panel: expanded views of characteristic vibrational bands—amide I (C=O stretch, ~1650 cm^−1^), amide II (N-H bend, ~1550 cm^−1^), and carboxylate (COO^−^, ~1400 cm^−1^). Right panel: expanded region of O-H/N-H stretching vibrations (~3200–3600 cm^−1^), showing peak broadening consistent with increased hydrogen bonding in Bio-GelMA. (**g**) ^1^H nuclear magnetic resonance (^1^H NMR) spectra (D_2_O, 400 MHz) of NHS-PEG12–biotin, GelMA, and Bio-GelMA. Arrows highlight diagnostic proton signals: PEG methylene protons (δ ≈ 3.6 ppm), biotin methyl protons (δ ≈ 1.3 ppm), and GelMA backbone protons (δ ≈ 7.8–8.2 ppm); spectral superposition confirms covalent incorporation of NHS-PEG12–biotin onto GelMA side chains. (**h**) Photocrosslinking behavior: rapid sol–gel transition of 10% (*w*/*v*) GelMA and Bio-GelMA solutions upon exposure to 405 nm UV light for 15 s, confirming preserved photoreactivity after biotinylation. (**i**) Representative cross-sectional scanning electron microscopy (SEM) images of lyophilized 10% (*w*/*v*) GelMA and Bio-GelMA hydrogels, revealing comparable porous microarchitectures with interconnected pore networks. *** *p* < 0.001. Reproduced from Reference [64] with permission from the American Chemical Society. Copyright © 2023, American Chemical Society.

To maintain the biological activity of EVs under harsh conditions in vivo (low pH, high temperature, and enzymatic degradation), scientists introduced a biotin-modified GelMA (BioGelMA) to deliver EVs. BioGelMA successfully underwent a sol–gel transition after 15 s of 405 nm UV light irradiation (Figure 5a–i) [64]. Previous studies have explored the binding of EVs to GelMA hydrogel systems. However, knowledge of the exact effects of these systems on cell migration and angiogenesis in the field of wound healing remains deficient. Doshi et al. demonstrated precise control of cell migration through modulation of the properties of GelMA hydrogels, which may play an important role in angiogenesis that promotes wound healing [65].

Recently, the delivery of EV mimics has also been investigated in the field of photosensitive hydrogels. For the first time, researchers have applied engineered EV mimics to skin wound healing and tissue regeneration, effectively addressing the hindrance of low EV production to clinical use. GelMA offers better efficiency in the delivery of EVs and EMs, with improved topical delivery concentration through slow release [66].

##### pH-Responsive Hydrogels

Unlike light and temperature, which were previously mentioned as factors affecting sol–gel transformation, pH-responsive hydrogels are more likely to affect drug release in delivery systems used for EVs. Wang et al. developed an injectable thermoresponsive and pH-responsive hydrogel FHE with multifunctional properties, including injectability, self-repairing, antimicrobial, and adhesion properties. This multifunctional hydrogel consisted of PF-127, oxidized hyaluronic acid (OHA), and EPL, which were formed by a reversible Schiff base reaction between OHA and EPL. EVs can be released in a weakly acidic environment due to the breakage of Schiff base bonds [67]. This pH-responsive release is particularly relevant for acute wounds, which often exhibit an acidic microenvironment.

##### Multi-Stimuli-Responsive Hydrogels

Despite improved therapeutic efficacy, single-stimuli-responsive hydrogels are still often unsatisfactory due to intrinsic drawbacks. Most current hydrogels do not possess multifunctional properties that make them ideal drug delivery systems for wound healing/skin reconstruction [68]. Wang et al. reported an injectable adhesive temperature-sensitive multifunctional polysaccharide dressing (FEP) with continuous pH-responsive EV release. The injectable and adhesive properties of this hydrogel provide good maneuverability and do not detach from the wound during the healing process. These biomedical features may enhance its high capability in angiogenesis and wound healing [59].

Li et al. developed a dual-sensitive hydrogel loaded with exosomes derived from hUCMSCs. The hydrogel was constructed using genipin crosslinking, which provides structural integrity and stability. The figures showed that the hydrogels had the highest dissolution rate at 35 °C and pH 6.5 compared to other temperature and pH conditions. These exosomes were encapsulated within the hydrogel matrix to facilitate their sustained release and therapeutic effects on wound healing [69]. α-Lipoic acid (LA) is a hydrophobic compound with outstanding antioxidant properties, reducing cellular damage induced by oxidative stress [70]. LA was incorporated into the chitosan skeleton to develop a photocrosslinkable chitosan hydrogel, which does not require additional photoinitiators. It can achieve H_2_O_2_-, glucose-, and pH-responsive release. This multifunctional hydrogel possesses the ability to control the release of exosomes and regulate the wound microenvironment, effectively providing the necessary therapeutic support [71]. Currently, there is a trend towards environmental stimuli-responsive hydrogels that can sense multiple external stimuli simultaneously.

##### Conductive Hydrogels

Endogenous electric fields naturally occur at the wound site upon epithelial disruption, generating a guiding electrical cue for cellular migration (electrotaxis) [72]. To effectively mimic and enhance these native electrical signals, conductive hydrogels must possess electrical conductivity that aligns with the physiological range of human skin. The electrical conductivity of intact human skin typically ranges from 10^−4^ to 10^−2^ S/cm, while wound exudate and deeper dermal tissues can exhibit slightly higher conductivity (up to 10^−1^ S/cm) due to the presence of ions and biomolecules [73,74]. Therefore, incorporating conductive polymers, such as polypyrrole, polyaniline and PEDOT, or conductive nanomaterials, such as graphene oxide and carbon nanotubes, into hydrogel matrices aims to elevate the material’s conductivity into this physiological range (10^−3^ to 10^−1^ S/cm) [75]. This matching of conductivity is critical for restoring the transepithelial potential (TEP) and accelerating the migration of keratinocytes and fibroblasts toward the wound center.

Therefore, increasingly, researchers are proposing to incorporate conductive materials into hydrogel dressings to improve the wound healing process. Zhang et al. incorporated multiwalled carbon nanotubes exhibiting good conductivity into the hydrogels, creating a stable three-dimensional structure for EVs and metformin loading [76]. The conductive properties allow the hydrogel to mimic the electrical conductivity of biological tissues, facilitating cellular electroactivity and signaling and helping to accelerate the wound healing process.

#### 4.1.3. Critical Material Properties and Release Kinetics of Hydrogel–EV Composites

The therapeutic efficacy of EV-loaded hydrogels is highly influenced by the physicochemical properties of the hydrogel matrix, which must be precisely engineered to dynamically adapt to the evolving wound microenvironment. These critical parameters include crosslinking chemistry, gelation kinetics, mechanical modulus, degradation behavior, and EV retention and release profiles (Table 1).

Regarding crosslinking, hydrogels are typically formed via physical interactions, such as thermal gelation, ionic interactions and host–guest recognition, or chemical reactions, such as photocrosslinking, Schiff base reactions and enzymatic crosslinking. For in situ delivery, injectable hydrogels formed through rapid chemical crosslinking, such as Schiff base reactions between aldehyde-modified and amine-containing polymers, or physical crosslinking, such as temperature-responsive chitosan or Pluronic F-127, are preferred, with an ideal gelation time ranging from seconds to a few minutes to ensure easy injection into irregular defects and rapid solidification that prevents leakage while conforming to the wound bed [77]. The mechanical modulus should closely mimic the native extracellular matrix of skin tissue to facilitate cellular mechanotransduction, proliferation, and migration, with G’ typically ranging from 100 Pa to 10 kPa depending on polymer concentration and crosslinking density, thereby preventing collapse under wound contraction forces while providing a compliant microenvironment that does not restrict regeneration [78]. Wet-tissue adhesiveness, a critical feature given that wound exudate creates a highly hydrated environment, is engineered through catechol groups, such as dopamine-modified hyaluronic acid or gelatin and mimicking mussel adhesive proteins, or dynamic covalent chemistry (e.g., dynamic Schiff base bonds), which form hydrogen, covalent, or coordination interactions with tissue proteins even in the presence of water, ensuring robust hemostatic and sealing capabilities [79]. The degradation profile must be synchronized with the wound healing timeline, which is typically 1–3 weeks for acute wounds and longer for chronic ones, and occurs primarily via hydrolysis (cleavage of ester bonds) or enzymatic degradation, such as matrix metalloproteinases degrading gelatin or collagen, with a well-designed rate ensuring that the scaffold provides initial structural support and is subsequently replaced by newly synthesized ECM without triggering a foreign body response. Finally, the primary function of the hydrogel matrix is to prevent rapid wash-out of EVs by wound exudate, enhancing local retention; the release kinetics are typically governed by a combination of diffusion and matrix degradation, with an initial burst release of surface-located EVs via diffusion followed by sustained release as the polymer network gradually degrades, enlarging the mesh size and allowing diffusion of deeply encapsulated EVs. The retention efficacy is highly dependent on crosslinking density and polymer concentration. A tighter mesh restricts diffusion but must be balanced against potential EV inactivation.

**Table 1:** Physicochemical properties and release kinetics of representative EV-loaded hydrogels for wound healing [79,80,81].

Hydrogel Matrix	Crosslinking Type	Gelation Time	Modulus (G’)	Wet Adhesion Mechanism	Degradation Time	EV Release Profile
GelMA	Photocrosslinking (UV)	~30 s	~2–5 kPa	N/A (physical fit)	14–21 days (enzymatic)	Sustained (degradation-dominant)
OHA/CMCS	Schiff base (chemical)	~15 s	~800 Pa	Dynamic Schiff base	10–14 days (hydrolytic)	Biphasic
DA-Gelatin	Physical/Chemical	~120 s	~1.2 kPa	Catechol–amine	7–10 days (enzymatic)	Sustained
PF-127	Thermo-reversible	~10 s	~1.0 kPa	N/A	3–5 days (dissolution)	Burst (diffusion-dominant)

### 4.2. Other Delivery Systems

**Figure 6:**
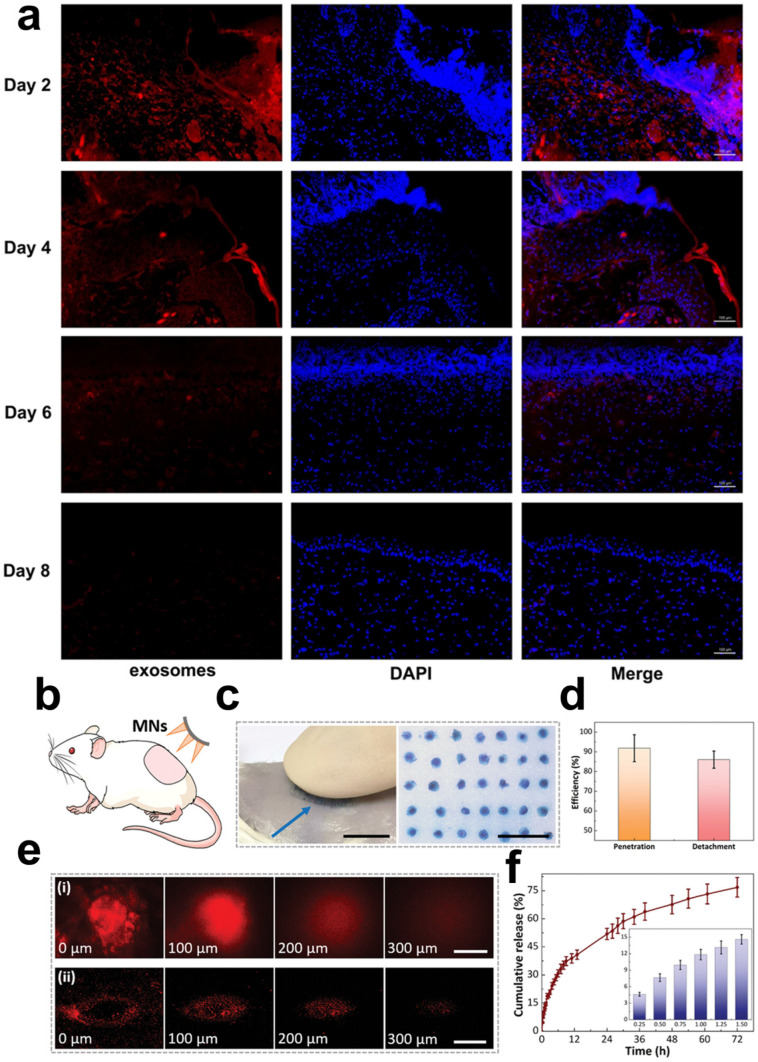
(**a**) Immunofluorescence staining of dorsal skin tissue harvested from mice treated with GelMA/PEGDA@T+exos MN patches at post-application time points of 2, 4, 6, and 8 days. Nuclei were counterstained with DAPI (blue); collagen I (green) and CD31 (red) were co-immunolabeled to assess extracellular matrix deposition and angiogenesis. Scale bar: 100 μm. (**b**) Schematic illustration of the in vivo application protocol: indwelling microneedles were vertically pressed into the shaved dorsal skin of mice using controlled axial force, followed by substrate detachment while retaining needle tips within the dermis. (**c**) Representative optical images documenting microneedle insertion: (left) microneedle patch pressed onto mouse dorsal skin; (right) excised skin showing blue-dyed microneedle tips embedded in the dermis. Arrows indicate retained needle structures. Scale bars: 5 mm (overview), 1.5 mm (magnified inset). (**d**) Quantitative assessment of microneedle performance in murine skin: penetration efficiency (percentage of needles successfully inserted into the dermis) and substrate detachment efficiency (percentage of needles remaining in skin after patch removal), determined from *n* = 6 independent animals. (**e**) Confocal fluorescence microscopy images visualizing the spatial distribution of red fluorescent nanoparticles (NPs) released from indwelling microneedle tips into dorsal skin at depths of 0, 100, 200, and 300 μm after 6 h: (**i**) skin with intact needle tips in situ; (**ii**) skin following complete needle tip removal. Nuclei were stained with DAPI (blue). Scale bar: 200 μm. (**f**) In vitro cumulative release kinetics of red fluorescent nanoparticles from P7L2DMA microneedle tips into PBS (pH 7.4) over 72 h (*n* = 6). Inset: Initial burst release phase within the first 1.5 h. MN: microneedle. MN: microneedle. Panel (**a**) reproduced from Reference [62] with permission from Springer Nature under the terms of the Creative Commons CC BY license. Panel (**b**–**f**) reproduced from Reference [85] with permission from John Wiley and Sons.

In addition to hydrogels, other biomaterials have been explored for EV delivery in wound healing, though they are less extensively studied. Microneedle patches (MNPs) represent a significant new method for targeted administration of EVs. Traditional exogenous drug therapy relies on repeated local injections, which can cause secondary damage. Hydrogel MNPs are used as biomaterials for delivering EVs due to their superior biocompatibility, minimally invasiveness, extended drug retention time and high loading efficiency. Due to the hydrophilic nature of hydrogels, hydrogel MNPs absorb mesenchyme and dissolve when inserted into the skin [82]. Hydrogel MNPs should be made of materials that are biocompatible and have better dissolution properties. Researchers effectively fabricated MNPs utilizing micro-molding technology, incorporating GelMA and polyethylene glycol diacrylate for the controlled release of endothelium-derived exosomes. These MNPs have demonstrated successful application in skin tissue regeneration in diabetic C57BL mice. Immunofluorescence imaging revealed that within 8 days, notable degradation of PKH26-labeled EVs facilitated their efficient penetration into the damaged skin areas on the backs of diabetic mice, thereby fostering angiogenesis and promoting epithelialization (Figure 6a) [62]. Zeng et al. encapsulated the M2 macrophage-derived EVs in the needle tip and polydopamine (PDA) nanoparticles in the backing layer to simultaneously inhibit inflammation at the wound site and improve angiogenesis. In vitro, released MEs increased macrophage polarization towards the M2 phenotype [83].

Polyvinyl alcohol (PVA) can form physically or chemically crosslinked networks, due to the abundant hydroxyl groups [84]. Zhang et al. present an adjustable indwelling hydrogel microneedle that can be used for diabetic wound healing. The microneedle consists of a PVA hydrogel that is ion-responsive due to the Hofmeister effect, so that the stiffness of the resulting microneedle tip can be modulated by sulfate ions to ensure skin penetration and release of exosomes (Figure 6b–f) [85]. This regulatory strategy targeting the local microenvironment provides a highly promising avenue for the treatment of diabetic ulcers by activating endogenous cellular responses and significantly improving local physiology.

### 4.3. Discussions and Translational Perspectives of Extracellular Vesicle Delivery Platforms

The clinical translation of EV-based wound therapies relies heavily on selecting an optimal delivery platform that aligns with the specific pathological characteristics of the target wound. While conventional hydrogels, stimuli-responsive hydrogels, and MNPs all facilitate localized EV retention, their distinct physicochemical properties confer unique advantages and limitations suited for different clinical scenarios (Table 2).

Conventional hydrogels, such as HA, alginate, and chitosan-based, offer excellent biocompatibility, cost-effectiveness, and straightforward manufacturing processes. Their inherent hydrophilicity maintains a moist wound environment and absorbs exudates, making them ideal for mild to moderate acute wounds with standard exudation. However, their primary limitations lie in the lack of precise spatiotemporal control over EV release and susceptibility to rapid degradation in highly proteolytic chronic wounds. Consequently, they often require frequent administration, which increases the risk of secondary injury and patient non-compliance.

Stimuli-responsive hydrogels, such as thermoresponsive, photosensitive, and pH-responsive hydrogels, represent an advanced evolution designed to overcome the passive release drawbacks of conventional systems. Their primary advantage is the capacity for “on-demand” or microenvironment-triggered EV release, which maximizes drug utilization and minimizes systemic loss. For instance, pH-responsive systems are particularly advantageous for chronic diabetic wounds characterized by acidic microenvironments and chronic inflammation. Photosensitive hydrogels (e.g., GelMA) offer rapid in situ gelation that perfectly conforms to irregular deep wounds. Nevertheless, their clinical application is currently hindered by complex fabrication procedures, potential cytotoxicity of photoinitiators or synthetic polymers, and stringent storage requirements. Scalability and reproducibility also remain significant bottlenecks for industrial manufacturing.

MNPs provide a distinct mechanism of action via minimally invasive transdermal delivery. Their major advantage lies in their ability to bypass the stratum corneum barrier and deliver EVs directly into the deeper viable epidermis or dermis, making them exceptionally suited for targeted therapies such as scar reduction or treating superficial chronic ulcers. MNPs also offer the benefit of painless self-administration, improving patient compliance. However, their limitations include relatively low EV loading capacity per patch and restricted total fluid absorption, rendering them less suitable for highly exudative or heavily infected wounds requiring robust biobarrier protection. Furthermore, achieving controlled, long-term release (beyond a few weeks) remains technically challenging for hydrogel-forming MNPs.

In summary, there is no universal delivery system for all wound types. Future clinical implementation will likely demand combinatorial strategies, such as integrating the robust barrier function of conventional hydrogels with the precise targeting capability of MNPs, or engineering multi-stimuli-responsive systems that simultaneously address infection, oxidative stress, and EV release. A rational design, tailored to the specific phase and etiology of the wound, is the critical step toward personalized precision regenerative therapy.

## 5. Conclusions and Perspectives

This review highlights EV-based therapies, particularly when integrated with LDDSs, as promising next-generation tools for wound healing. By systematically summarizing the molecular mechanisms across all healing phases and evaluating the design rationales of various delivery platforms, from conventional hydrogels to advanced stimuli-responsive systems and microneedle patches, we have underscored how LDDSs empower EVs by maximizing local bioavailability, preserving bioactivity, and providing structural guidance for tissue regeneration. Despite remarkable preclinical successes, the clinical translation of EV-LDDS hybrids faces several formidable challenges that must be critically addressed: (i) scalable manufacturing and quality standardization. A primary translational bottleneck is the inefficient and difficult-to-scale production of EVs. Traditional 2D cell culture yields are insufficient for industrial mass production. Future research must prioritize the optimization of 3D culture systems, hollow-fiber bioreactors, and scalable isolation techniques (e.g., tangential flow filtration) to ensure high-yield, GMP-compliant manufacturing. Furthermore, the lack of standardized quality control metrics, encompassing EV size, surface marker expression, cargo potency, and absence of contaminants, hinders reproducibility. The establishment of unified characterization guidelines, aligned with frameworks such as MISEV, is urgently required, including (ii) in vivo fate, stability, and pharmacokinetics. While LDDSs significantly improve local retention, the precise in vivo biodistribution, degradation pathways, and half-life of EVs released from these biomaterials remain poorly understood. Advanced in vivo tracking technologies and comprehensive pharmacokinetic/pharmacodynamic profiling are needed to optimize dosing regimens and ensure biosafety. (iii) Regulatory pathways and clinical trial design. EV-LDDS hybrids represent complex combination products involving both biological entities and medical devices. This dual nature creates ambiguity in regulatory classification (e.g., as biologics, medical devices, or combination products). Close collaboration between researchers and regulatory agencies is essential to establish specific evaluation criteria for safety, efficacy, and quality tailored to these hybrid systems.

Looking forward, the field is poised for transformative advancements along several promising research directions. Firstly, future LDDSs should evolve from passive or single-stimuli-responsive carriers to intelligent systems capable of real-time monitoring of the wound microenvironment. By integrating sensing elements that detect specific biomarkers, such as pH, glucose levels, or inflammatory cytokines, these platforms could dynamically adjust EV release profiles in response to real-time healing progress. Then, the therapeutic efficacy of EV-LDDS can be further amplified through combination with other therapeutic modalities. Co-delivering EVs with growth factors, antimicrobial peptides, gene editing tools, such as CRISPR/Cas9, or oxygen-generating nanomaterials may yield synergistic effects that address the multifaceted pathology of chronic wounds simultaneously. Finally, as our understanding of EV heterogeneity deepens, future therapies may utilize patient-specific or wound-specific EV formulations. Tailoring the EV source and the LDDS physicochemical properties to the specific etiology and phase of the wound, such as diabetic foot ulcers and severe burns, will be pivotal for personalized regenerative therapy.

In conclusion, while significant hurdles remain, the continuous maturation of EV biology, biomaterial engineering, and manufacturing technologies is steadily bridging the gap between bench and bedside. By addressing these translational challenges and embracing multidisciplinary innovations, EV-LDDS hybrids hold immense potential to revolutionize the clinical landscape of precision wound therapy.

## Figures and Tables

**Table 2:** Summary and comparison of representative EV delivery systems for wound healing [50,51,52,53,54,55,56,57,58,59,60,61,62,63,64,83,84].

Delivery System	Material	Crosslinking Method	EV Source	Release Time	Advantages	Limitations	Potential Clinical Applications
Conventional hydrogels	Hyaluronic acid derivatives	Schiff base reaction (in situ)	M2 Macrophages	Up to 21 days	Excellent antibacterial effect (eradicates 99% MRSA); sustained release; good biocompatibility.	Lacks responsiveness to dynamic wound microenvironment; may require precise preparation.	Infected wounds; chronic wounds with high bacterial burden.
	Alginate	Ionic crosslinking (divalent cations)	Adipose-derived stem cells	~172 h	Low cost; high biocompatibility; mild gelling conditions; reduces scar formation.	Uncontrolled passive release; weak mechanical strength.	Acute full-thickness skin wounds; minimizing scar formation.
	Chitosan (CEC/DCMC)	Schiff base reaction	Bone marrow mesenchymal stem cells	Sustained release	Inherent antimicrobial and hemostatic properties; modulates inflammatory microenvironment.	Poor water solubility of unmodified chitosan; mechanical fragility.	Diabetic wounds; highly exudative wounds.
Stimuli-responsive hydrogels	PF-127 (Thermoresponsive)	Physical crosslinking (temperature)	Human umbilical cord mesenchymal stem cells	Continuous/Sustained	Injectable; liquid at low temp and gel at body temp; FDA-approved base material.	Concentration-dependent phase transition temperature; potential rapid erosion.	Chronic diabetic wounds; irregular wound beds requiring injectable scaffolds. Chronic diabetic wounds; irregular wound beds requiring injectable scaffolds.
	GelMA/BioGelMA; GelHA (Photosensitive);	Photopolymerization (UV/Visible light)	Adipose-derived stem cells (hypoxia-pre-treated)	Prolonged retention	Rapid in situ gelation; tunable mechanical properties; adapts to irregular defects.	Potential cytotoxicity of photoinitiators; limited penetration depth of UV light.	Deep irregular wounds; diabetic wounds requiring enhanced angiogenesis.
	Genipin-crosslinked hydrogel	Chemical crosslinking (genipin)	Human umbilical cord mesenchymal stem cells	Dual-sensitive (temp/pH) sustained release	Dual sensitivity to temperature and pH; high structural integrity and stability.	Genipin crosslinking kinetics must be strictly controlled to avoid over-rigid gels.	Full-thickness cutaneous wounds requiring long-term stable scaffolding.
Microneedle Patches	GelMA/PEGDA	Photopolymerization	Human umbilical vein endothelial cells	~8 days (in vivo degradation)	Minimally invasive; bypasses stratum corneum; sustained delivery to deep skin layers.	Low loading capacity; limited exudate absorption capability.	Diabetic wounds; promoting epithelialization and localized angiogenesis.
	PGLADMA/GelMA	Photopolymerization	Human mesenchymal stem cells	Up to 3 weeks (30% released)	Core–shell structure for coordinated prolonged release; high drug retention.	Complex micro-molding fabrication; difficult to scale up mass production.	Scarless skin regeneration; long-term localized anti-inflammatory therapy.
	PVA (Ion-responsive)	Physical crosslinking (Hofmeister effect)	Mesenchymal stem cells	Sustained (3 days profiled)	Adjustable stiffness for skin penetration; indwelling needle tips for continuous release.	Requires precise control of sulfate ion concentration; tip detachment may occur.	Diabetic ulcers requiring precise depth penetration and localized microenvironment regulation.

## Data Availability

No new data were created or analyzed in this study.
